# Targeted pre-partum strategies to suppress *Toxocara vitulorum* hypobiotic larvae: Reducing transmission to calves and genotypic insights into buffalo infections

**DOI:** 10.14202/vetworld.2025.329-340

**Published:** 2025-02-13

**Authors:** Reem M. Ramadan, Alaa M. Wahby, Noha Mohamed Bakry, Hend M. Auda, Faten F. Mohammed, Mohamed M. El-Bahy, Sahar Hussein Abdalla Hekal

**Affiliations:** 1 Department of Parasitology, Faculty of Veterinary Medicine, Cairo University, 12211, Giza, Egypt; 2 Department of Natural Resources, Faculty of African Postgraduate Studies, Cairo University, 12613, Giza, Egypt; 3 Department of Veterinary Infectious and Medicine, Faculty of Veterinary Medicine, Cairo University, 12211, Giza, Egypt; 4 Department of Pathology, College of Veterinary Medicine, King Faisal University, Al-Ahsa, 31982, Saudi Arabia

**Keywords:** buffalo infections, *COX-1* gene, fenbendazole, scanning electron microscopy, strategic treatment, *T. vitulorum*

## Abstract

**Background and Aim:**

*Toxocara vitulorum* infections in lactating buffaloes pose significant health and economic challenges due to maternal transmission of inhibited larvae to calves via colostrum and milk. This study aimed to identify *T. vitulorum* species morphologically and genetically and to evaluate a novel strategic treatment using fenbendazole to suppress larval transmission.

**Materials and Methods:**

Morphological and genetic characterization of *Toxocara* species was performed using light and scanning electron microscopy and mitochondrial *COX-1* gene analysis. Pregnant buffaloes previously infected with *T. vitulorum* were administered fenbendazole (10 mg/kg body weight) 15 days before parturition (dbp). The animals were divided into three groups based on the interval between treatment and parturition: 6 days (G-1), 10 days (G-2), and 15 days (G-3). Colostrum, milk, and fecal samples were collected to assess larval and egg counts, respectively.

**Results:**

The genetic analysis confirmed the species as *T. vitulorum* with 100% nucleotide similarity to reference sequences. The treatment effectively suppressed larval transmission in G-1, with no larvae detected in colostrum or milk, and significantly reduced larval counts in G-2 and G-3. Fecal egg counts of treated buffaloes and their calves were markedly lower than untreated controls. Statistically significant reductions in worm burden were observed, particularly in the group treated 6 dbp.

**Conclusion:**

A single dose of fenbendazole administered 6 dbp effectively interrupted the *T. vitulorum* transmission cycle, reducing larval presence in colostrum and milk and minimizing worm burdens in buffaloes and calves. Morphological and molecular analyses highlighted the efficacy of *COX-1* gene markers in species identification and phylogenetic studies. This strategic intervention represents a practical approach to controlling *T. vitulorum* infections, improving herd health, and reducing environmental contamination.

## INTRODUCTION

*Toxocara vitulorum* is a significant ascarid nematode with a global distribution that primarily infects the small intestine of large ruminants, such as cattle and buffaloes [1]. This parasite poses a substantial threat to livestock health, especially in young calves, where it can lead to severe clinical manifestations, including diarrhea, colic, anemia, emaciation, intestinal obstruction, and, in extreme cases, death. These infections cause considerable economic losses, particularly in regions where water buffaloes are raised for milk, meat, and leather production. Therefore, understanding the epidemiology and control of *T. vitulorum* is crucial for improving livestock productivity and farm profitability [2, 3].

Adult bovines acquire *T. vitulorum* infection through the ingestion of eggs containing second-stage larvae. However, patent infections in adult animals are rare due to the unique life cycle of the parasite. The larvae migrate to tissues such as the liver, muscles, and lymph nodes, where they become dormant (hypobiotic or inhibited) [4, 5]. Reactivation of arrested larvae occurs shortly before parturition, typically between 1 and 8 days before calving. During this critical period, larvae migrate to mammary glands, where they can be transmitted to suckling calves through colostrum, milk, or even amniotic fluid [6]. This mode of transmission leads to heavy parasite burdens in newborn calves, with infection rates peaking in the first 2 days postpartum (dpp) and gradually declining within 10–18 days [7].

The absence of parasitic eggs in the feces of adult buffaloes often misleads farmers into believing that the animals are parasite-free, even though they harbor inhibited larvae capable of reactivating and infecting their offspring [8]. This cycle perpetuates the spread of *T. vitulorum*, leading to significant challenges in controlling the infection and its associated economic impacts.

Despite the availability of various anti-nematodal drugs, most studies by have Taha *et al*. [9], Sarani and Hataminejad [10] focused on treating newborn calves after infection rather than targeting the inhibited larvae in pregnant buffaloes. Taha *et al*. [9] have primarily explored the efficacy of drugs such as ivermectin (IVM), doramectin (DRM), and fenbendazole against adult worms or calves after infection. However, Sarani and Hataminejad [10] have investigated a strategic treatment approach aimed at the critical reactivation period of inhibited larvae in pregnant buffaloes before parturition. This approach can prevent maternal transmission and substantially reduce infection rates in calves.

In addition to therapeutic strategies, accurate identification of *T. vitulorum* species through morphological and molecular characterization is essential for devising effective control measures [11]. Advances in DNA-based techniques, particularly polymerase chain reaction (PCR) amplification of nuclear and mitochondrial genetic markers, offer precise tools for assessing genetic diversity, phylogenetic relationships, and drug sensitivity in parasitic nematodes [12]. The mitochondrial *COX-1* gene is particularly highly conserved in *Toxocara* species, making it an excellent molecular marker for species identification and phylogenetic studies.

This study addresses the gap in the strategic control of *T. vitulorum* by focusing on inhibited larvae during the critical reactivation phase in pregnant buffaloes. The objectives were to (1) identify the *Toxocara* species causing maternal infections in buffaloes through morphological and molecular analysis, (2) evaluate the efficacy of a single dose fenbendazole treatment administered approximately 15 days before parturition (dbp) in eradicating reactivated larvae, and (3) assess the impact of this treatment on reducing larval transmission to calves. By targeting the inhibited larvae at a critical stage, this study proposes a novel strategy to break the infection cycle, ultimately reducing the parasite burden in both buffaloes and their offspring.

## MATERIALS AND METHODS

### Ethical approval

The Institutional Animal Care and Use Ethical Committee (IACUC-VET-25122023825) of the Faculty of Veterinary Medicine, Cairo University, Egypt, authorized all study procedures.

### Study period and location

The study was conducted from January to December 2022 on a private farm in Giza Province, Egypt (31.0°E, 29.0°N). Data collection covered the period from two months before parturition through early lactation. Monthly fecal examinations were performed before the drying phase to assess parasite egg counts and treatment effectiveness.

### Lactating buffalo farms in Egypt

Lactating buffalo farms in Egypt are primarily small- to medium-sized private enterprises, following a local breeding and production system, as described by Fahim and Abdel-Salam [13]. The buffalo population in Egypt has reached an average of 3.9 million heads [14]. The animals are typically fed a balanced diet consisting of dry roughage (straw), green roughage (clover and local grass), and concentrate (soybean, wheat bran, and cottonseed meals) at an average intake of 10.90 ± 2.85, 4.98 ± 2.89, and 1.51 ± 0.80 kg dry matter per day per head, respectively [13]. Artificial insemination is performed between 40 and 60 dpp, depending on the condition of the uterus. Milk is collected throughout a lactation period of 260–285 days, with an average milk yield per animal ranging from 1310 kg to 1450 kg. The calving interval is typically 400–415 days. Special care and isolation are provided to females and their calves during the first 3 months after parturition. Newborn calves continue suckling until they are 2–3 months old. Milk collection ceases 2 months before the subsequent parturition, as the buffaloes enter late pregnancy [15]. This 2-month period is the primary period during which drug administration in milk is permitted. According to regulations, selling meat or milk from treated animals is prohibited for 2–4 weeks post-treatment (wpp). During this dry period, little attention is given to inhibited larvae, such as those of *T. vitulorum* and other nematodes, unless they are endemic to the farm. Farm owners typically administer specific anti-parasitic drugs to their animals randomly during this period.

### Animal selection

A total of 180 lactating buffaloes (*Bubalus bubalis*) diagnosed with *T. vitulorum* infection were selected. The diagnosis was confirmed by detecting parasite eggs in fecal samples from both the buffaloes and their calves using the concentration flotation method, as reported by El-Naggar *et al*. [16]. Calf mortality was recorded at two months of age. The farm owner had been administering broad-spectrum antiparasitic drugs, including IVM, DRM, and moxidectin (MXD), at their recommended therapeutic doses. Despite treatment, *T. vitulorum* eggs persisted in the feces, with counts ranging between 100 and 500 eggs per gram (epg), as determined by the McMaster technique. Infected females were identified with ear tags and monitored throughout pregnancy. The buffaloes were housed in semi-open barns under standard conditions management conditions, with communal access to feeding and watering areas. Their diet consisted of berseem clover (*Trifolium alexandrinum*), corn silage, and a commercial concentrate mixture formulated to meet their nutritional requirements. Routine vaccination and deworming protocols were maintained during the study.

### Collection and identification of worms

During previous observations, some calves exhibited severe diarrhea and subsequently died. Postmortem examination of these calves revealed a heavy infestation of *Toxocara* species adult worms in their intestines. Sufficient samples of the worms extracted from the intestines of slaughtered calves were collected and immediately transferred under ice preservation to the Parasitology Laboratory at the Faculty of Veterinary Medicine, Cairo University, for morphological and genotypic identification. The extracted worms were placed in clean Petri dishes and washed with normal saline [17]. The anterior and posterior ends of the worms were excised and then fixed in alcohol-glycerin to prepare permanent mounts, following the procedure described by Biswas *et al*. [18]. Both living active worms and mounted specimens were used for identification, as previously described by Chelladurai *et al*. [1].

### Scanning electron microscopy (SEM)

According to Salem *et al*. [19], two active, motile worm samples from each slaughtered, infected calf were selected, washed 3 times with warm phosphate-buffered saline (pH = 7.4), and then preserved in 2.5% glutaraldehyde. The ascarids were dehydrated using an upgraded ethanol series ranging from 50.0% to 100.0%. The nematodes were completely dried using an Autosamdri-815 (Tousimis, Germany) CO_2_ critical point dryer. After slicing the anterior and posterior ends, the nematodes were attached to 20-nm gold-coated stubs. The nematodes were photographed using a scanning electron microscope (JSM 5200, electron probe) and a microanalyzer (Jeol, Japan).

### DNA extraction, amplification, and sequencing

DNA was extracted from two worms collected from each infected calf, and milk samples were collected from the mothers using the QIAamp Fast DNA Tissue kit (Cat. No. 51404) following the manufacturer’s instructions (Qiagen, Germany) [20]. DNA was then eluted using 50 µL of elution buffer. Conventional PCR procedures were used for DNA amplification according to previously described methods by Salem *et al*. [21]. The PCR reaction was performed using a Taq PCR Master Mix kit (Cat. No. 201445) (Qiagen) to amplify a fragment of 708 bp and the Platinum™ PCR Super Mix High Fidelity kit (Cat. No. 12532016) (Thermo Fisher Scientific, USA) to amplify a fragment of 15 Kbp.

As described by Sultan *et al*. [22], the analysis was conducted using the forward primer sequence 5’ GGTCAACAAATCATAAAGATATTGG 3’ and the reverse primer sequence 5’ TAAACTTCAGGGTGACCAAAAAATCA 3’ for the amplification of the mitochondrial *COX-1* gene. PCR was performed under the following conditions: initial denaturation at 94°C for 2 min, followed by 40 cycles of denaturation at 94°C for 1 min, annealing at 45°C for 1 min, and extension at 72°C for 1 min. A final termination step at 72°C for 5 min was performed to amplify 708-bp fragments. The PCR amplicons were identified using electrophoresis on a 2% agarose gel in a TBE solution containing ethidium bromide (0.5 g/mL), which allowed their detection under ultraviolet light. The PCR amplicons were quantified using an Invitrogen Life Technologies Qubit® Fluorometer (Eugene, OR, USA) and purified using a PCR DNA and Gel Band Purification kit (GE Healthcare, Little Chalfont, Buckinghamshire, UK) [23].

PCR amplicons were sequenced using a BigDye® Terminator Cycle Sequencing kit (Thermo Fisher Scientific) on an Applied Biosystems Genetic Analyzer sequencer with the original forward and reverse primers used in the first PCR experiment. The assembled sequences were compared to and aligned with other relevant sequences deposited in GenBank using the Basic Local Alignment Search Tool program, accessible at (https://blast.ncbi.nlm.nih.gov/Blast.cgi). The nucleotide sequences obtained during this study were submitted to GenBank. The edited *COX-1* sequences were uploaded to GenBank to obtain accession numbers (OQ933098). Phylogenetic trees based on nucleotide sequences were generated using MEGA 11 (https://www.megasoftware.net/) and the Maximum Likelihood method [24].

### Control strategy

#### The proposed strategy base

The animals that previously harbored *T. vitulorum* eggs in their feces were reexamined 1 month before the expected time of parturition, which was designated as day 0. Animals without any other parasitic infections were selected for the study. These animals were isolated in January 2022 to evaluate the suggested strategic treatment, as most re-migrating larvae are collected and transmitted through colostrum/milk to suckling calves during the first 2 dpp [6]. The previously tissue-inhibited larvae must be reactivated and migrate toward the uterus shortly before parturition. Therefore, the suggested strategy aimed to apply one treatment with an effective larvicidal drug, such as fenbendazole at a dosage of 10 mg/kg body weight (b.w.), 2 weeks before the estimated time of parturition for each pregnant buffalo. This treatment kills migrating larvae before they arrive in the fetus and udder [8]. The period from treatment to the actual occurrence of parturition was recorded to assess the efficacy of this approach in eradicating re-migrating L4 larvae. The impact of this strategic treatment on the mean number of larvae present in colostrum/milk, as well as epg of the parasite in the mothers before and after parturition, was evaluated, along with the effects in calves during the first 2 months of their lives, in comparison with similar non-treated control groups.

### Experimental design

The study involved 90 *Toxocara*-infected pregnant buffaloes (4–8 years old) with known insemination dates and confirmed parasitologically through fecal examination. The buffaloes were divided into two groups of 45 animals each: the treated and control groups. The treated group received a single oral dose of Panacur® (Merck, USA) (10 mg/kg) 15 days before the estimated parturition date, while the control group received no treatment [25, 26]. The treated group was further subdivided based on the actual time of parturition post-treatment as follows: 6 days (G-1, n = 10), 10 days (G-2, n = 12), and 15 days (G-3, n =1 4). All animals were housed under standardized farm conditions and received balanced diets with roughage, concentrates, and ad libitum access to water. Hygiene protocols were strictly followed, and animals were monitored daily to minimize external variables that could affect parasitic activity or drug efficacy. Parturition timing was estimated using insemination dates and was confirmed by monitoring physiological changes, such as udder enlargement, pelvic ligament relaxation, and vaginal secretion. Daily colostrum/milk and fecal samples were collected from all animals following treatment and throughout the observation period. At parturition, mothers and calves were isolated in separate pens until weaning (2 months post-parturition), with balanced rations and water provided *ad libitum*. The collected samples were analyzed for infection rates, larval counts per milliliter of colostrum/milk, and epg of feces.

### Parasitological examination of the samples

After visual examination of all collected fecal samples to detect whole worms or parts of worms, the samples were examined for various diagnostic stages of suspected parasites. The concentration flotation technique was used to diagnose small eggs and protozoan oocysts, as described in previous studies by Ramadan *et al*. [27] and Ramadan *et al*. [28]. The surface layer was transferred onto glass slides and examined microscopically. Subsequently, further inspection of the samples was conducted using the concentration sedimentation technique, which involved sedimentation after centrifugation at 300× *g* for 3 min in distilled water to diagnose large-sized eggs, as outlined by Mahdy *et al*. [29]. The sedimented layer was examined microscopically using the procedure described above. The diagnosed eggs were measured using micrometer slides, and the mean size was calculated. The mean epg was determined for each sample using the McMaster egg counting technique, as previously described by Sultana *et al*. [30].

### Examination of milk samples

Early morning and before calf suckling, 50 mL of sterile colostrum/milk samples were collected in clean, dry tubes, transferred to the laboratory in an ice bath, and stored in a refrigerator to ensure that the examination was as fresh as possible. According to the technique described by Urhan *et al*. [31], each sample was mixed with distilled water at a ratio of 1:4 in a 250 mL centrifuge tube and then centrifuged at 3000× *g* for 20 min using a cooling centrifuge (4°C). The samples were kept at 4°C for 2 h to allow settling; the fat collected on the top surface of the tube was then removed. The supernatant was then withdrawn, and the sediment was divided into two additional centrifuge tubes (15 mL capacity) [32]. This sediment was centrifuged again, as before, after removing the supernatant. The sediment was thoroughly mixed and examined as a thin film under a microscope at low and high magnification (400×). All diagnosed larvae were counted, and the number of larvae per 1.0 mL of milk was calculated [33].

### Statistical analysis

The data were analyzed using Statistical Package for the Social Sciences version 28 (IBM Corp., Armonk, NY, USA) [34, 35] to assess the efficacy of fenbendazole treatment in reducing *T. vitulorum* larvae and egg counts. Descriptive statistics, including means and standard deviations, were calculated for larval counts in colostrum/milk and egg counts in feces across the treated and control groups. Comparative analyses were performed using paired-sample t-tests to evaluate differences in larval and egg counts within groups over time [36]. Independent-sample t-tests assessed differences between treated and untreated groups. One-way analysis of variance (ANOVA) was used to determine statistical differences among the subgroups (G-1, G-2, and G-3), based on the timing of treatment relative to parturition, followed by Tukey’s post hoc test for pairwise comparisons. Statistical significance was set at p ≤ 0.05. Longitudinal data, such as changes in larval counts over the observation period, were analyzed using repeated measures ANOVA to identify trends and interactions between time points and treatment effects. Effect sizes (Cohen’s d) were computed to quantify the magnitude of differences observed. All statistical outcomes were reported with corresponding p-values, confidence intervals, and degrees of freedom, where applicable, ensuring transparency and reproducibility. Graphs and tables were utilized to present the findings, highlighting key differences in larval and egg counts across treatment groups and time points. The parameters are presented in the form of mean ± 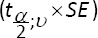. The standard error is calculated as,


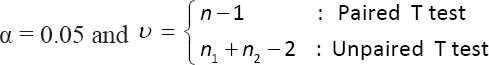


The degrees of freedom were recorded according to p-value “p,” and the statistical significance is ≥ the obtained critical value 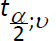 of a statistic (T). The obtained results are considered extremely significant if p ε [0; 0.0006], very statistically significant if p ε [0.0006; 0.001], statistically significant if p ε [0.001; 0.05], not quite statistically significant if p ϵ [0.05; 0.08], or non-significant when p ϵ [0.008; ∞].

## RESULTS

### Morphological identification of adult worms

**Figure 1:**
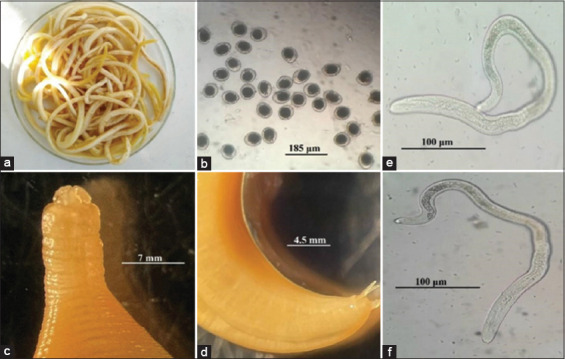
(a) *Toxocara vitulorum* collected from buffaloes and calves, (b) *T vitulorum* eggs, (c) anterior end of the worm showing lips, (d) posterior end of the male showing spicules, and (e and f) *T vitulorum* larvae with tapered tails.

Macroscopic and microscopic identification of fresh and mounted specimens of *Toxocara* adult worms extracted from the intestines of freshly slaughtered calves originating from this farm confirmed that they were *T. vitulorum*. The fresh worms are translucent and whitish in color and have a cylindrical wall. The mean sizes of males and females ranged from 17.56 to 23.2 mm in average length and 0.5–0.65 mm in width, respectively. The anterior end is characterized by small, broad lips at the base and narrow anteriorly, with three lips divided into three sections (Figure 1). The body does not significantly taper toward extremities. The esophagus is 2.8–4.3 mm long and has a posterior granular ventriculus. The male’s tail typically forms a small spike-like appendage with approximately five pairs of post-cloacal papillae, with the anterior pair being large and double. The number of pre-cloacal papillae is variable. The spicules measure 0.89–1.23 mm in length. The vulva is located approximately one-eighth of the body length from the anterior end. Eggs extracted from the proximal part of the female uterus, close to the female genital opening, are sub-globular in shape, with a mean size of 70–92 × 57–70 µm. These eggs are covered by a non-operculated shell externally coated with a thick, finely pitted albuminous layer containing immature one-cell stages (Figure 1).

## SEM

**Figure 2:**
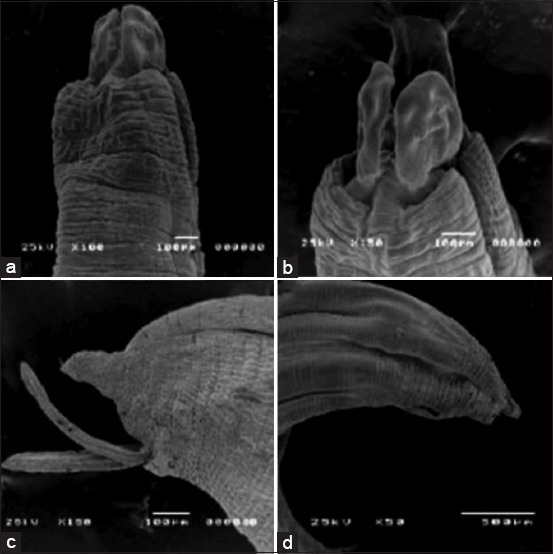
Scanning electron micrographs of adult *Toxocara vitulorum* body surface (selected picture): (a) the anterior end of the worm showed lips and annulated cuticle, (b) the anterior end of *T. vitulorum* showed three prominent lips, one dorsal and two sub ventral, (c) posterior end of male showed two spicules, and (d) the posterior end of the female worm showed a straight and short tail end.

SEM analysis of the previously obtained adult worms revealed smooth, non-segmented body walls covered by smooth cuticles characterized by fine corrugations that give rise to a series of characteristic transverse striations (annulations). These form continuous rings around the body (annuli) at regular intervals, creating a false segmented appearance. The annuli are separated by small grooves known as sub-annuli (Figure 2a). The parasite features three well-defined lips surrounding the mouth opening: two subvental and one dorsal lip. Each lip has a pair of sensory papillae on its surface and a single dentigerous ridge composed of a line of minute unicuspid denticles (Figure 2b). The male posterior end exhibits two spicules, post-cloacal papillae, and a bell-shaped projection (Figure 2c). In contrast, the female posterior end is distinguished by a straight, short tail, which reveals the anal opening (Figure 2d).

### Molecular identification

**Figure 3:**
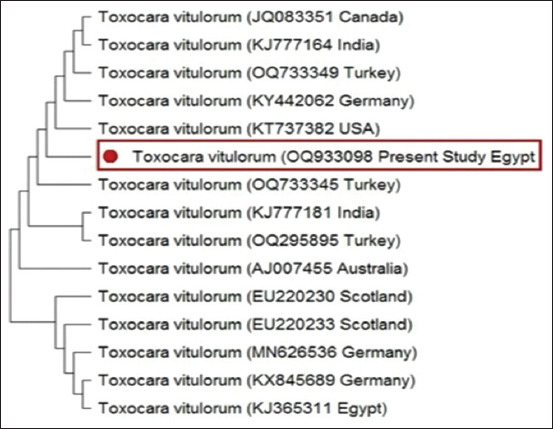
Phylogenetic analysis of *Toxocara vitulorum* based on *COX-1* regions. The trees were constructed using the maximum likelihood method. The GenBank accession numbers for the strains are provided, and the sequences from this study are indicated by a filled red circle.

Mitochondrial *COX-1* gene analysis amplified a 708-bp product from *T. vitulorum* samples. Sequence comparison revealed 100% nucleotide identity with *T. vitulorum* GenBank accessions KY442062 and OQ733340 and 99.82% identity with KT737382 while showing significantly lower similarity with *Toxocara canis* (89.49%) and *Toxocara* *cati* (87.3%). Phylogenetic analysis using the maximum likelihood method revealed a distinct clustering of *T. vitulorum* sequences that were clearly distinguished from *T. canis* and *T. cati*. This clustering corroborates the genetic uniqueness of *T. vitulorum* and its evolutionary divergence from other *Toxocara* species (Figure 3).

### Effect of the recommended strategic treatment on inhibited larvae and adult worms

#### Effects of strategic treatment on larval counts in colostrum and milk

According to the actual time of parturition in treated and control calves, ten animals gave birth to their calves after 6 days post-treatment (dpt), 12 animals after 10 dpt, and 14 animals after 15 dpt. Daily investigations and calculations of the mean number of larvae per milliliter of colostrum/milk indicated that no larvae could be diagnosed in animals treated at 6 dbp, in contrast to similar control untreated females. Larvae were gradually diagnosed in the milk of the other two groups treated with 10–15 day before treatment and in the control untreated group, as described in Table 1. In the treated group, larvae were diagnosed at only 2 dbp and remained present in the milk until the 14^th^ dpp. In the untreated group, larvae were diagnosed earlier (at 6 dbp) and persisted until the 18^th^ dpp. The mean number of larvae per mL of colostrum/milk was lower in the treated groups (1.3 ± 0.13 larvae/mL) than in the untreated group (3.37 ± 0.18 larvae/mL) (Table 1).

**Table 1:** Variations in the mean number of *Toxocara vitulorum* larvae/ml milk from treated and untreated buffaloes during the entire observation period.

Animal groups (n = 20)	Duration of shedding from	Time of maximum shedding	Mean number over the whole period
From treated Buffalo	2–14 dpp	2^nd^-dpp	1.3 ± 0.13 larva/1 mL
From the untreated one	6–18 dpp	2^nd^-dpp	3.37 ± 0.18 larva/1 mL

dpp=Days postpartum, dpt=Days post-treatment

Statistical analysis of the difference in the mean number of larvae per milliliter of colostrum/milk between treated and untreated females (Table 1a) revealed a complete absence of larvae in the milk of females treated at 6 dbp. This indicated a significant statistical difference in the calculated value using paired sample statistics, with a critical value 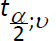 for this group compared with the similar untreated group. The mean difference was 9.514. A slightly lower difference was observed in the mean number of larvae in animals that gave birth after 10 dpt, with a value of 6.913; however, this difference remained statistically significant. The difference was significant when comparing the group that gave birth after 15 dpt with the mean number of larvae per mL of milk in the control untreated females (Table 1a).

**Table 1a:** T-value for the difference in the mean *Toxocara vitulorum* larvae/ml milk during the whole observation period (15^th^ dbp till complete disappearance of larvae in milk) between treated and untreated buffaloes.

The group born their calves after	Mean larvae/mL milk	The difference was considered to be
6 dpt	9.514	Extremely statistically significant
10 dpt	6.913	Very statistically significant
15 dpt	4.286	Statistically significant

([Paired Samples Statistics] α = 0.05 and n = 10 and 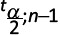
=1.793), dpt=Days post-treatment, dbp=Days before parturition

#### Effect of strategic treatment on the number of egg/gram feces in adult buffaloes

The applied strategic treatment (Table 2) resulted in a marked decrease in the mean number of inhibited larvae developing into adult worms in the intestines of treated and untreated buffaloes. This was demonstrated by variations in the mean number of epg of feces and duration of infection in treated and control buffaloes. No *T. vitulorum* eggs were detected in the feces of the buffalo group treated at 6 dbp, whereas eggs were detected in the other two groups of females treated at 10–15 dbp and in the control group. The periods of egg shedding in feces were 4–10 wpp in treated females and 3–14 wpp in untreated females. This was associated with a decrease in the total mean epg of feces in both groups, which was 630 ± 24.4 epg in treated females and 3866 ± 34.8 epg in untreated females (Table 2).

**Table 2:** Variations in the mean number of *Toxocara vitulorum* mean eggs/gram feces shed from treated and untreated buffaloes during the observation period.

Animal groups (n = 20)	Duration of shedding	Time of maximum shedding	Mean number along the period
Treated ♀*	4–10 wpp	6 wpp	630 ± 24.4 epg
Un treated ♀	3–14 wpp	8 wpp	3866 ± 34.8 epg

wpp=Weeks post-treatment, epg=Eggs per gram, *This infection was recorded in ♀ that born their calves at the 10^th^ and 15^th^ days post-treatment

Statistical analysis of the difference in mean epg of feces between treated and untreated females (Table 2a) revealed the absence of *T. vitulorum* eggs in the feces of animals treated on the 6^th^ day. This indicates a significant statistical difference in the calculated value using paired sample statistics, with a critical value 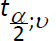 for this group compared with similar untreated females. This evolution can be attributed to the high mean difference of 9.715. The difference was statistically significant when comparing the groups treated at 10 dbp with a value of 6.112 A difference of 3.856 was observed in animals that gave birth after 15 dbt, which still holds a significant value compared with the mean epg in untreated control females (Table 2a).

**Table 2a:** T-value for the difference in the mean number of *Toxocara vitulorum* eggs/gram in feces of treated and untreated buffaloes during the whole observation period (15^th^ dbp till complete disappearance of eggs).

The group born their calves after	Mean eggs/gram	The difference is considered to be
6 dpp	9.715	Extremely statistically significant
10 dpp	6.112	Very statistically significant
15 dpp	3.856	Statistically significant

([Paired Samples Statistics] α = 0.05 and n = 10 and 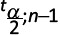
=2.053), dbp=Days before parturition, dpp=Days postpartum

#### Effect of strategic treatment on the number of egg/gram feces

Investigating the effect of the timing of treatment on infection and the level of worm burden, which reflects the duration of shedding and the mean epg of feces in newly born calves from treated and control females (Table 3), we found no parasite eggs in the feces of calves born from females treated at 6 dbp. In contrast, eggs were detected in calves born from females treated at 10–15 dbp and in the control group. The feces of calves born from treated females contained a gradually increasing number of *T. vitulorum* eggs for 4–11 weeks of age, whereas this period was longer (3–14 weeks) in calves born from untreated females. Moreover, the mean epg of feces from infected calves born to treated females was lower (677 ± 8.1 epg) than that recorded in calves born to untreated control females (7602 ± 12 epg) (Table 3).

**Table 3:** Variations in the mean number of *Toxocara vitulorum* mean eggs/gram feces of calves born from treated and untreated animals during the whole observation period.

Animal groups (n = 20)	Duration of shedding from	Time of maximum shedding	Mean number along the period
Calves from treated ♀*	4–11 wpp	7 weeks old	677 ± 8.1 epg
Calve from untreated ♀	3–14 wpp	6 weeks old	7602 ± 12 epg

wpp=Weeks post-treatment, epg=Eggs per gram, * This infection was recorded in calves born from ♀ treated at 10 and 15^th^ days before parturition

Among the observations, morbidity symptoms, including severe diarrhea, colic, and emaciation, were recorded in calves born from untreated mothers and were not observed in any calves born from treated females. In general, the appearance of larvae in milk or *T. vitulorum* eggs in feces began at a low mean and increased gradually post-treatment or parturition, with specific peaks at different times, as demonstrated in Tables 1-3.

Statistical analysis of the difference in mean fecal epg between calves born from treated and untreated females (Table 3a) showed that no *T. vitulorum* eggs could be diagnosed in the feces of calves born from females treated at 6 dbp compared with calves born from untreated females. This difference was highly statistically significant in the calculated value using paired sample test statistics, with a critical value 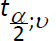 for this group. This evolution is attributed to the high mean difference of 8.611. The difference between the groups of calves born from treated mothers at 10 dbp was calculated to be statistically significant compared with those from untreated females, with a calculated difference of 5.552. The comparison of the last group of calves born from mothers treated at 15 dbp with those from untreated females resulted in a difference of 3.675, which is considered significant (Table 3a).

**Table 3a:** T-value for the difference in the mean number of *Toxocara vitulorum* eggs/gram in the feces of calves born from treated and untreated buffaloes during the observation period (11 weeks old).

The group born from mothers treated at	Mean eggs/gram	The difference was considered to be
6 dbp	8.611	Extremely statistically significant
10 dbp	5.552	Very statistically significant
15 dbp	3.675	Statistically significant

([Paired Samples Statistics] α = 0.05 and n = 10 and 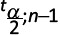
=2.067), dbp=Days before parturition

## DISCUSSION

Water buffalo breeding is a crucial aspect of animal husbandry, and these animals are raised in many regions for meat, milk, and leather production. Raising healthy and productive calves is essential for sustainable and profitable water buffalo farming. *T. vitulorum* is a pathogenic nematode that infects cattle and water buffaloes, particularly young animals, leading to significant economic losses, particularly in newborn buffalo calves [31]. Although water buffaloes are generally less susceptible to many diseases than cattle, they are highly vulnerable to *T. vitulorum* infections, worsening these losses [37]. Morphological identification of the isolated *Toxocara* species in the present using light and SEM analysis revealed that they align with previous descriptions of *T. vitulorum* as they have a reliable characterization of the species. The body morphology, including the translucent, whitish cylindrical shape, non-tapering extremities, and distinct tripartite lips, corroborates the findings of Chelladurai *et al*. [1]. The measured sizes of males (17.56–23.2 mm length, 0.5–0.65 mm width) and females, along with the esophagus length (2.8–4.3 mm) and posterior granular ventriculus, are consistent with observations reported by Biswas *et al*. [18]. The male’s spike-like tail with five pairs of post-cloacal papillae and spicule length (0.89–1.23 mm) agrees with the detailed morphology documented by Dewair and Bessat [4]. Similarly, the egg characteristics, including the sub-globular shape (70–92 × 57–70 µm) and the pitted albuminous shell containing immature one-cell stages, align closely with those reported by Fouad *et al*. [17].

This study used mitochondrial *COX-1* gene analysis to precisely identify *Toxocara* species at the species level. The obtained sequence exhibited 100% nucleotide identity with *T. vitulorum* GenBank accessions KY442062 and OQ733340 and 99.82% identity with KT737382. In contrast, it exhibited significantly lower similarity to *T. canis* (89.49%) and *T. cati* (87.3%). These results highlight the high conservation of *COX-1* expression and its utility in distinguishing *T. vitulorum* from closely related species. Phylogenetic analysis using the maximum likelihood method revealed distinct clustering of *T. vitulorum* sequences, which were clearly separated from those of *T. canis* and *T. cati*. This clustering confirms the genetic uniqueness and evolutionary divergence of *T. vitulorum*. These findings underscore the reliability of *COX-1* markers in molecular taxonomy and phylogenetic studies of the *Toxocara* genus. Furthermore, phylogenetic analysis identified clustering patterns associated with geographic origin, reflecting intraspecific genetic diversity. The Egyptian isolate (OQ933098) clustered closely with those from Turkey (OQ733345) and India (KJ777181), suggesting shared evolutionary trajectories and potential transmission pathways. In contrast, isolates from geographically distant regions, such as Australia (AJ007455) and Scotland (EU220230), formed distinct branches, indicating significant genetic divergence. These findings are in agreement with previous studies by Chelladurai *et al*. [1], Chen *et al*. [12], Sultan *et al*. [22], and Urhan *et al*. [31] and highlight the importance of integrating morphological and molecular approaches for a comprehensive understanding of *T. vitulorum* diversity and epidemiology, as adopted in the present study.

Several attempts have been made to control adult *T. vitulorum* infections in cattle and calves using various anti-nematodal drugs. For example, Goossens *et al*. [6] investigated IVM and DRM, while Avcioglu and Balkaya [38] evaluated the efficacy of IVM, DRM, and MXD in naturally infected calves. Given the existing knowledge regarding the efficacy of these drugs against nematodes, fenbendazole was selected for the present study due to several factors. First, the animals on this farm were considered resistant to IVM, DRM, and MXD due to repeated, random use. This was supported by the mild but persistent presence of eggs of *T. vitulorum* in the feces of these animals during the observation period before implementation of the strategic control plan. Moreover, no specific data were found regarding the efficacy of these drugs against inhibited larvae. By contrast, according to Williams *et al*. [8], fenbendazole has a proven effect against inhibited muscle larvae, as demonstrated by a study comparing muscle larvae counts in treated and control animals after slaughter using a digestion technique.

This study demonstrated the efficacy of a single dose of fenbendazole in reducing *T. vitulorum* larval transmission from lactating buffaloes to their calves. Statistical analysis revealed significant reductions in larval presence, particularly in the group treated 6 dbp (G-1), in which larvae were completely absent from both colostrum and milk (p ≤ 0.0001). In contrast, groups treated 10 and 15 dbp (G-2 and G-3) showed significant reductions in larval counts, with p-values of 0.01 for both groups, indicating less effective, though still beneficial, treatment outcomes. These results suggest that earlier treatment timing maximizes the drug’s biological effectiveness, with diminishing returns as the interval between treatment and parturition increases. A similar approach was previously reported by Stuedemann *et al*. [7], who used fenbendazole (5 mg/kg) to treat adult *T. vitulorum* in buffaloes and 28-day-old calves without targeting the inhibited larvae.

The differences in treatment efficacy can be attributed to the timing of fenbendazole administration relative to the reactivation and migration of larvae. *T. vitulorum* larvae re-enter the migratory phase shortly before parturition, traveling to the mammary glands where they are transmitted to the calves. Early fenbendazole administration likely disrupted larvae before migration, whereas later treatments allowed more larvae to reach the udder, resulting in higher numbers detected in milk and feces. This process also shortened the duration of larval shedding. Early treatment resulted in the complete cessation of larval shedding in milk, whereas later treatment reduced the shedding period compared with that in untreated animals. This reduction minimizes environmental contamination because fewer eggs and larvae are released into the pastures and the environment. This strategy could reduce parasite load on farms, improving health outcomes for both adult buffaloes and calves. The absence of larvae in colostrum and milk following early treatment could significantly lower the risk of infection in newborn calves, reducing morbidity and improving herd productivity. By shortening the shedding period, the strategy also reduced the potential for reinfection in treated buffaloes, thereby reducing the need for repeated anthelmintic treatments, which may contribute to drug resistance. Complementary environmental control measures, such as manure management and rotational grazing, can further enhance this strategy by preventing the buildup of *T. vitulorum* eggs in the environment.

The study’s findings on egg shedding support previous research by Tamire and Beredo [39], which reported heavy worm burdens in infected calves, leading to high egg counts (8,000–100,000 epg). The reduced mean epg in treated calves aligns with Stuedemann *et al*. [7], however, it contrasts with Roberts [40], and Martin and Robertson [41], who suggested that decreasing infection rates and epg over time may be due to either the absence of larvae in milk or the development of resistance to new infections. Consistent with Goossens *et al*. [6], larvae appeared in colostrum/milk by 2 dbp, and *T. vitulorum* larvae typically reached the udder between 1 dbp and 8 dbp, remaining present in milk until 10–18 dpp. Transmission from cow to calf occurred in all calves within the first 2 days after birth, decreasing to 53% by day 6, 10% by days 8–9, and 2% after day 10, as reported by Roberts [40].

The results of this study demonstrated statistically significant differences between the treated and control groups. In G-1, larvae were completely absent from colostrum and milk (T-value: 9.514, p ≤ 0.0001), effectively preventing transmission to calves. In G-2 and G-3 groups, larval loads were reduced by >60% (T-value: 6.913) and 40% (T-value: 4.286), respectively (p ≤ 0.01), with shorter durations of larval shedding compared to untreated groups. The complete absence of larvae in G-1 highlights the highest efficacy of early treatment, whereas G-2 and G-3 showed diminishing but meaningful effects as treatment timing became less optimal. These reductions were correlated with improved calf health, as G-1 calves exhibited no morbidity symptoms, unlike those from untreated groups. Treated buffaloes also had significantly lower fecal egg counts, with a mean count of 630 epg in G-1 compared to 3866 epg in the control group. This reduction implies decreased environmental contamination by *T. vitulorum* eggs, thereby lowering the risk of pasture reinfection. Early treatment disrupted larval migration to the mammary glands, emphasizing the importance of timing when preventing transmission. These findings are consistent with those of previous studies by Abdel-Rahman *et al*. [42], Ashraf [43], and Schoener *et al*. [44], demonstrating the relationship between statistically significant reductions in larval burden and measurable improvements in animal health and environmental management.

The long-term benefits of this treatment strategy are promising, as it can help reduce the overall parasite burden in the herd and minimize transmission to future generations of calves. By targeting inhibited larvae shortly before parturition, fenbendazole disrupts the transmission cycle, potentially improving long-term herd health. However, potential limitations include the risk of reinfection due to environmental exposure and the possibility of partial resistance developing over time with repeated fenbendazole use in the same herd. Long-term studies are needed to monitor reinfection rates, larval dynamics, and the development of immunity in treated buffaloes to fully understand the sustainability of this approach.

## CONCLUSION

This study demonstrates the efficacy of a single-dose fenbendazole treatment administered 6 dbp in significantly reducing *T. vitulorum* larval transmission from buffaloes to their calves. Morphological and molecular analyses confirmed the species as *T. vitulorum*, with the *COX-1* gene serving as a reliable molecular marker for species identification and phylogenetic studies. The treatment achieved complete eradication of larvae in colostrum and milk in the G-1 group (treated 6 dbp) and substantial reductions in larval and egg counts in G-2 (10 days) and G-3 (15 days). Treated calves exhibited lower fecal egg counts, reduced morbidity, and improved health outcomes compared to those born to untreated mothers. These findings highlight the importance of strategic timing in mitigating the maternal transmission cycle and improving herd productivity. The study addresses a critical gap in *T. vitulorum* control by targeting inhibited larvae in pregnant buffaloes rather than focusing solely on infected calves. The integration of morphological, molecular, and phylogenetic analyses ensured accurate identification of *T. vitulorum*, providing a robust foundation for the findings. The treatment protocol demonstrated both immediate benefits in larval transmission suppression and long-term potential to reduce environmental contamination and herd infection rates. However, the study’s generalizability is limited as it was conducted under controlled conditions on a single farm, which may not fully represent the variability in farming practices and parasite dynamics across different regions. Furthermore, the impact of environmental management practices on reinfection rates and larval persistence was not thoroughly explored. Repeated use of fenbendazole could also lead to resistance, which requires ongoing surveillance. Large-scale studies across diverse farming systems and geographic locations are needed to validate and optimize the treatment protocol. Combining fenbendazole treatment with environmental and herd management strategies could further enhance control efforts. Long-term monitoring of drug efficacy and resistance patterns is essential to ensure sustainable parasite management. Expanded phylogenetic analyses of *T. vitulorum* populations across regions could uncover transmission dynamics and genetic diversity, informing targeted interventions. This strategic intervention provides a promising pathway to breaking the maternal transmission cycle of *T. vitulorum*, safeguarding buffalo health, and enhancing productivity. Further research is recommended to refine and expand the applicability of this approach under varying real-world conditions.

## DATA AVAILABILITY

All data generated or analyzed in this study are included in this published article.

## AUTHORS’ CONTRIBUTIONS

RMR and AMW: Collected samples, microscopic examination, and PCR analysis. FFM: Scanning electron microscopy. SHAH, MME, and HMA: Conceptualized and designed the study. RMR, NMB, SHAH, and MME: Experimental design, data collection, analysis, and interpretation, and drafted and reviewed the manuscript. All authors have read and approved the final manuscript.
